# A Novel Electrocardiographic Index for the Diagnosis of Diastolic Dysfunction

**DOI:** 10.1371/journal.pone.0079152

**Published:** 2013-11-05

**Authors:** Mehdi Namdar, Patric Biaggi, Barbara Stähli, Bernhard Bütler, Rubén Casado-Arroyo, Danilo Ricciardi, Moisés Rodríguez-Mañero, Jan Steffel, David Hürlimann, Christian Schmied, Carlo de Asmundis, Gian-Battista Chierchia, Andrea Sarkozy, Thomas F. Lüscher, Rolf Jenni, Firat Duru, Walter J. Paulus, Pedro Brugada

**Affiliations:** 1 Heart Rhythm Management Centre, Cardiovascular Division, UZ Brussel — VUB, Brussels, Belgium; 2 Cardiovascular Centre, Cardiology, University Hospital of Zurich, Zurich, Switzerland; 3 Department of Physiology, VU University Medical Center, Amsterdam, The Netherlands; 4 Service de Cardiologie, Hôpitaux Universitaires de Genève, Geneva, Switzerland; University of Cologne, Germany

## Abstract

**Background:**

Although the assessment of diastolic dysfunction (DD) is an integral part of routine cardiologic examinations, little is known about associated electrocardiographic (ECG) changes. Our aim was to investigate a potential role of ECG indices for the recognition of patients with DD.

**Methods and Results:**

ECG parameters correlating with echocardiographic findings of DD were retrospectively assessed in a derivation group of 172 individuals (83 controls with normal diastolic function, 89 patients with DD) and their diagnostic performance was tested in a validation group of 50 controls and 50 patients. The patient group with a DD Grade 1 and 2 showed longer QTc (422±24ms and 434±32ms vs. 409±25ms, p<0.0005) and shorter Tend–P and Tend–Q intervals, reflecting the electrical and mechanical diastole (240±78ms and 276±108ms vs. 373±110ms, p<0.0001; 409±85ms and 447±115ms vs. 526±119ms, p<0.0001). The PQ–interval was significantly longer in the patient group (169±28ms and 171±38ms vs. 153±22ms, p<0.005). After adjusting for possible confounders, a novel index (Tend–P/[PQxAge]) showed a high performance for the recognition of DD, stayed robust in the validation group (sensitivity 82%, specificity 93%, positive predictive value 93%, negative predictive value 82%, accuracy 88%) and proved a substantial added value when combined with the indexed left atrial volume (LAESVI, sensitivity 90%, specificity 92%, positive predictive value 95%, negative predictive value 86%, accuracy 91%).

**Conclusions:**

A novel electrocardiographic index Tend–P/(PQxAge) demonstrates a high diagnostic accuracy for the diagnosis of DD and yields a substantial added value when combined with the LAESVI.

## Introduction

 Diastolic dysfunction (DD) has gained much attention over the last decades and its assessment is nowadays not only an integral part of echocardiographic routine examinations but also of great importance in the evaluation of patients with dyspnea and/or heart failure symptoms [1]. As half of these patients are diagnosed with “diastolic heart failure” or “heart failure with preserved EF”, diastolic function and left ventricular filling pressures are put forward in the diagnostic work-up and differentiation from other causes [2]. Accordingly, relaxation of the cardiac muscle and its contributing factors have shifted to the center of scientific and clinical attention. While heart failure was mainly considered as a loss of contractile force for decades, progresses particularly in imaging technology have added much to the mechanistic understanding of DD as an underlying cause in many cases [1,3]. However, most noninvasive measurements of left ventricular relaxation, stiffness and filling pressures are indirect and not free of limitations, and are frequently based on simplified assumptions thereby limiting their general applicability. Moreover, their assessment is not infrequently highly variable in the same patient with changes in preload, afterload, and sympathetic tone, further complicating their measurement and interpretation. Finally, for a comprehensive assessment, co-factors such as age, heart rate, sex, body weight and blood pressure at the time of the examination have to be taken into consideration in order to obtain valid results [1–3]. 

In contrast, electrocardiographic (ECG) parameters are generally less prone to acute hemodynamic changes, show a great reproducibility and are operator-independent [4–6]. However, little is known about ECG changes in DD [7–9]. Our aim was therefore to investigate to investigate a potential role of ECG indices for the recognition of patients with DD.

## Methods

### Study population

 For the retrospective part of the study all individuals were included from the in-hospital echocardiographic database of the University Hospital of Zurich, Switzerland. Primary inclusion criterion of patients was a DD of any class according to the latest recommendations of echocardiographic assessment of DD as well as age > 18 years [1]. 

The same inclusion and exclusion criteria (see below) were applied for the validation part of the study, for which we recruited fifty consecutive unselected normal controls and patients, respectively. Interpretation of echocardiographic and ECG findings was performed analogous to the retrospective part of the study. However, a predefined ECG parameter, which was derived from the retrospective analysis, served for the determination of DD. Subsequently, the results were compared with the echocardiographic assessment, which served as a gold-standard and validation of the ECG parameters of interest. 

### Electrocardiographic parameters

**Figure 1:**
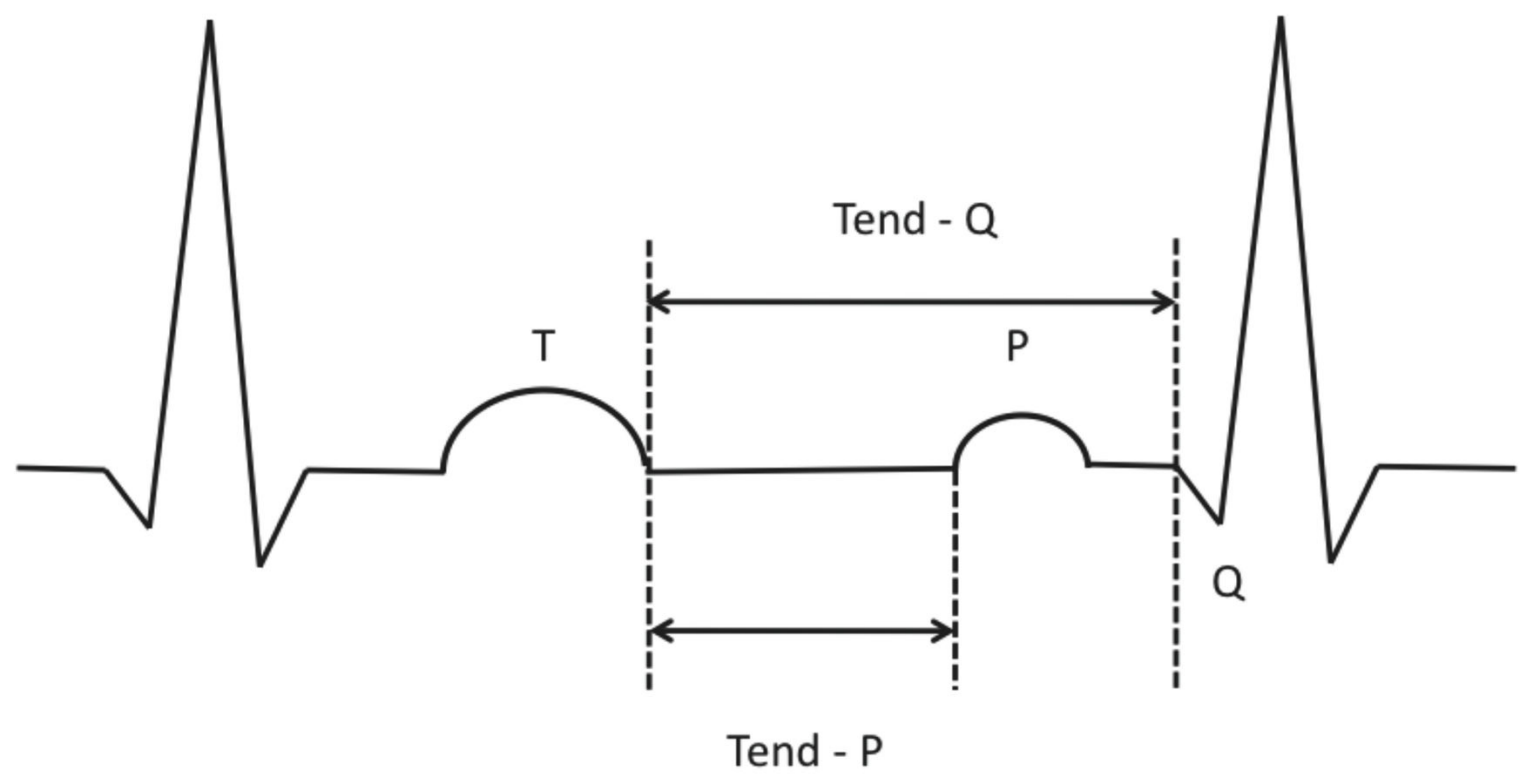
Schematic illustration of Tend-P and Tend-Q measurements. ECG-intervals of interest (Tend-P, Tend-Q) reflecting the mechanical diastole were both manually measured and calculated as: RR minus PQ minus QT for Tend-P and RR minus QT for Tend-Q.

 Twelve-lead surface ECG at initial diagnosis were independently analyzed by two experienced readers. Measurements were taken manually from the tracings at 25mm/sec. The observers were blinded to the echocardiographic findings, and the ECG reading has been performed by consensus reading. Standard criteria for ECG findings were applied: The QTc interval was calculated using the Bazett formula [10]. The QT/QTc dispersion was defined as the difference between the maximum and minimum QT/QTc interval of the 12 leads [11,12]. The Tpeak-Tend was measured in each precordial lead measured from the peak of the T-wave until the end of T-wave. In the case of negative or biphasic T waves, Tpeak was measured from the nadir of the T-wave. In accordance with the Lewis or Wiggers cycle, ECG-intervals of interest (Tend-P, Tend-Q, Figure 1) reflecting the mechanical diastole were also included in our analysis [13]. These two intervals were both manually measured (taking into account all ECG leads) and calculated as: RR minus PQ minus QT for Tend-P and RR minus QT for Tend-Q. Single leads with T waves smaller than 1.5 mm in amplitude were not included in the analysis. Patients were excluded if they had atrial fibrillation, higher than grade I AV-block, atrial and/or ventricular pacing and history as well as signs of acute ischemia and/or cardiopulmonary decompensation. 

### Echocardiography

All echocardiographic studies were performed and / or reviewed by experienced staff cardiologists. Specific loops and images for assessment of diastolic function were acquired according to the American and European guidelines of diastolic dysfunction [1] stored in DICOM format and later reviewed by two experienced echocardiographers blinded to the ECG parameters. As proposed by the guidelines, diastolic function was assessed using a combination of age, heart rate, left ventricular size and mass, indexed left atrial volume, pulmonary pressure estimate, mitral inflow pattern (E/A wave ratio, deceleration time), septal and lateral mitral annular tissue Doppler velocities (septal and lateral e’, respectively) and pulmonary vein flow pattern. Diastolic function was graded as normal or abnormal with impaired relaxation (grade 1), pseudonormal (grade 2) or restrictive pattern (grade 3). Values were given as mean of 3 heart beats in end-expiration. Throughout all echocardiographic findings, a consensus reading was again applied. Patients were excluded if they had poor echocardiographic image quality or poor quality tissue Doppler tracings, signs of left ventricular systolic dysfunction (EF < 55 %), regional wall motion abnormalities, pericardial effusion, severe valvulopathies including relevant annular calcification and suspected or known familiar forms of hypertrophic and/or infiltrative cardiopathies due to secondary ECG changes (i.e. T wave inversions, bundle branch blocks, ST segment changes), which would have otherwise falsified the interpretation of the indices of interest.

The study was approved and patient consent was waived by the local ethics committee of the University of Zurich (Kantonale Ethikkommission).

### Statistical analysis

Continuous variables were compared using the Student’s t-test or by ANOVA and are presented as means +/- standard deviation. Categorical variables were compared using Fisher’s exact test. Bland-Altman analysis was performed for the correlation of manually measured and calculated ECG-parameters. ROC analysis was performed to test for diagnostic performance of different ECG-parameters. Multivariate linear and logistic regression analyses were used to examine the independent association between ECG parameters of interest and the global assessment of DD. Analyses were performed using SPSS 17.0 software. A P value < 0.05 was considered statistically significant.

## Results

**Figure 2:**
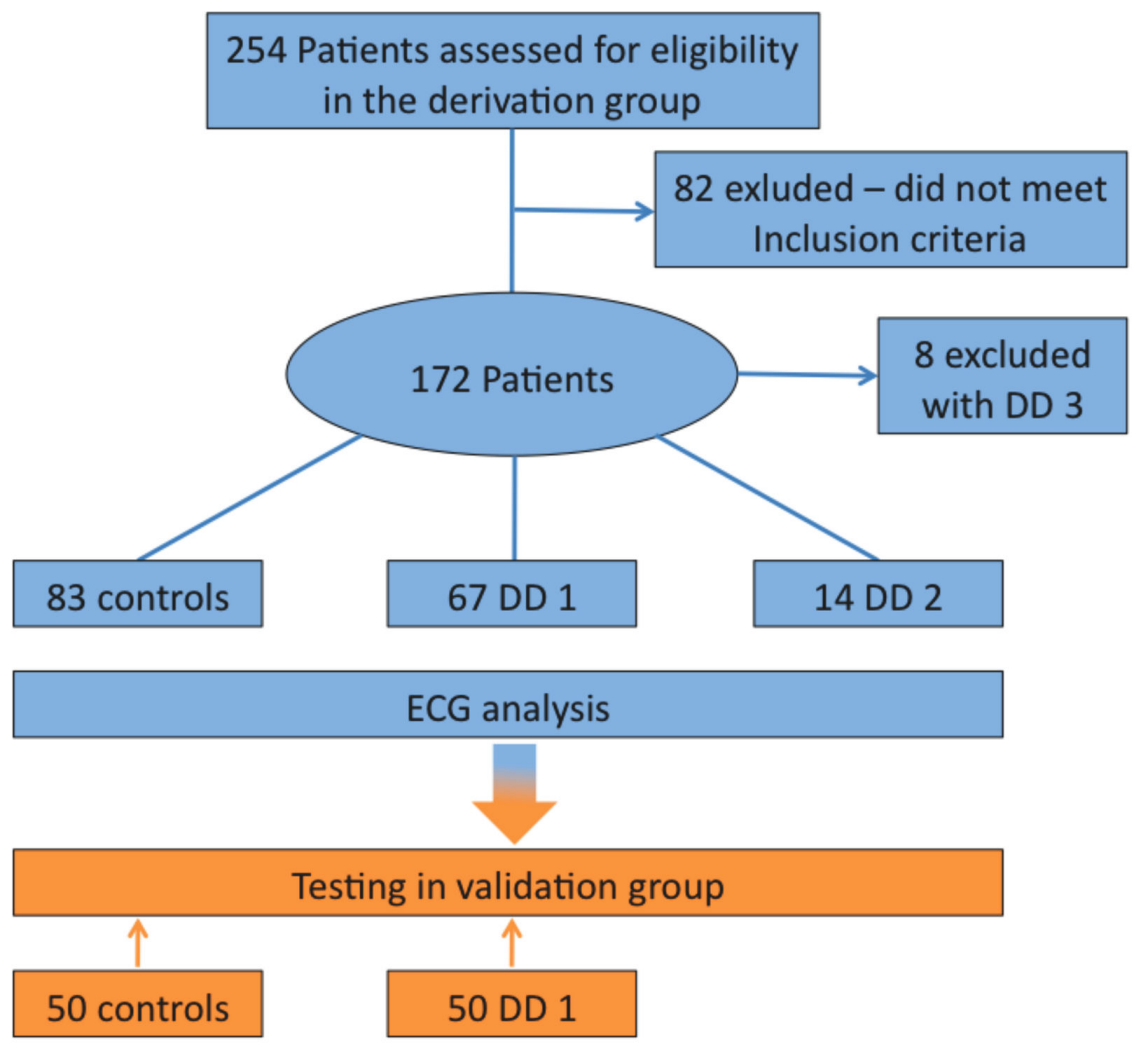
Patient flow chart.

 A total of 254 individuals were initially included. After application of the above-mentioned exclusion criteria, 172 (83 normal controls, 67 patients with DD grade 1, 14 patients with DD grade 2 and 8 patients with DD grade 3) remained for the analysis. However, all eight patients showing a restrictive left ventricular filling pattern (DD grade 3) had to be excluded from the study due to severe wall motion abnormalities along with severe left ventricular systolic dysfunction (Figure 2).

### Echocardiographic characteristics

Table 1 shows the demographic, clinical and echocardiographic characteristics of the different groups. Patients in the derivation group showing a DD Grade 1 and 2, respectively, were significantly older (67 ± 10 and 68 ± 11 vs. 48 ± 14 years, p<0.0001), had a higher indexed left atrial volume (33 ± 11 and 39 ± 11 vs. 23 ± 4 ml/m^2^, p<0.0001), a more pronounced septal (1.1 ± 0.2 and 1.2 ± 0.3 vs. 0.9 ± 0.2 cm, p<0.0001) and posterior (1.0 ± 0.1 and 1.0 ± 0.2 vs 0.9 ± 0.1 cm, p<0.0001) wall thickness and thus a higher left ventricular myocardial mass index (97 ± 30 and 97 ± 19 vs. 82 ± 20 g/m^2^, p<0.005). However, very few patients showed a “true” left ventricular hypertrophy as assessed by the left ventricular myocardial mass index. There were significantly more patients with a newly diagnosed arterial hypertension and diabetes mellitus both in the derivation and validation group, respectively. Of note, BNP levels were significantly higher in the patient population of both the derivation and validation group. As expected, Doppler-derived diastolic measurements differed significantly between controls and patients both in the derivation and validation group, respectively (Table 2). 

**Table 1:** Demographic, Clinical and Echocardiographic Characteristics of the derivation and validation group.

		**Derivation Group**				**Validation Group**	
**Parameter**	**Controls**	**DD 1**	**DD 2**	p-value*	**Controls**	**DD 1**	p-value*
Total number	83	67	14	-	50	50	-
Age (years)	48 ± 14	67 ± 10	68 ± 11	< 0.0001	31 ± 14	58 ± 8	< 0.0001
Male (n/%)	37 (45)	39 (58)	8 (57)	0.4	28 (56)	32 (64)	0.1
Body surface (m2)	1.85 ± 0.23	1.90 ± 0.24	1.86 ± 0.16	0.5	1.86 ± 0.45	1.88 ± 0.34	0.5
Potassium (mmol/ml)	3.9 ± 0.3	4.0 ± 0.4	4.2 ± 0.6	0.06	3.9 ± 0.4	3.9 ± 0.5	0.7
BNP (ng/l)	117 ± 125	584 ± 1082	1100 ± 1354	< 0.005	84 ± 30	445 ± 274	< 0.01
AHT (n/%)	7 (8)	30 (45)	12 (86)	< 0.001	3 (6)	27 (54)	< 0.0001
DM (n/%)	4 (5)	7 (10)	3 (21)	0.05	0 (0)	7 (14)	-
RV/RA (mmHg)	21 ± 5	24 ± 7	28 ± 10	< 0.01	24 ± 6	23 ± 7	0.5
LA ESVI (ml/m^2^)	23 ± 4	33 ± 11	39 ± 11	< 0.0001	23 ± 4	32 ± 3	< 0.0001
LV EDD (cm)	4.8 ± 0.5	4.7 ± 0.6	4.8 ± 0.6	0.4	4.7 ± 0.4	4.8 ± 0.7	0.5
SWth (cm)	0.9 ± 0.2	1.1 ± 0.2	1.2 ± 0.3	< 0.0001	0.9 ± 0.3	1.1 ± 0.3	< 0.0005
PWth (cm)	0.9 ± 0.1	1.0 ± 0.1	1.0 ± 0.2	< 0.0001	0.8 ± 0.2	1.0 ± 0.3	< 0.0005
LV EDV (ml)	96 ± 24	96 ± 27	96 ± 18	0.9	96 ± 11	95 ± 19	0.8
LVEF (%)	63 ± 4	63 ± 5	63 ± 4	0.7	65 ± 9	64 ± 8	0.7
LVEDVI (ml/m^2^)	52 ± 10	51 ± 12	52 ± 10	0.8	51 ± 11	52 ± 13	0.4
LVMMI (ml/m^2^)	82 ± 20	97 ± 30	97 ± 19	< 0.005	80 ± 19	95 ± 23	< 0.001
LVH male (n/%)	0	3 (4)	1 (7)	-	0	3 (6)	-
LVH female (n/%)	0	5 (7)	2 (14)	-	0	3 (6)	-
LVrTh	0.36 ± 0.05	0.42 ± 0.08	0.41 ± 0.10	< 0.0001	0.33 ± 0.04	0.41 ± 0.06	< 0.0001

DD 1 and DD 2 indicates diastolic dysfunction grade 1 and 2, * p value is given for the comparison of Controls vs DD (ANOVA in the derivation group), values are given as mean ± SD or numbers and percentages, AHT arterial hypertension, DM diabetes mellitus, RV right ventricle, RA right atrium, LA ESVI left atrial end-systolic volume index, LV left ventricle, EDD end-diastolic diameter, PWth posterior wall thickness, SWth septal wall thickness, LVEF left ventricular ejection fraction, LVEDVI left ventricular end-diastolic volume index, LVMMI left ventricular myocardial mass index, LVH left ventricular hypertrophy, LVrTh left ventricular relative thickness

**Table 2:** Doppler-echocardiographic parameters of diastolic dysfunction in the derivation and validation group.

		**Derivation Group**				**Validation Group**	
**Parameter**	**Controls**	**DD 1**	**DD 2**	p-value*	**Controls**	**DD 1**	p-value*
E/A ratio	1.27 ± 0.43	0.85 ± 0.47	1.39 ± 0.66	< 0.0001	1.40 ± 0.43	0.75 ± 0.11	< 0.0001
IVRT (ms)	91 ± 29	93 ± 25	89 ± 21	0.9	93 ± 19	94 ± 29	0.8
DT (ms)	189 ± 43	231 ± 61	195 ± 27	< 0.0001	188 ± 31	245 ± 58	< 0.0001
Septal e’ (cm/s)	8.4 ± 2.7	5.2 ± 2.3	4.6 ± 1.1	< 0.0001	8.1 ± 1.9	4.7 ± 1.7	< 0.0001
Lateral e’ (cm/s)	11.6 ± 3.4	8.0 ± 2.9	6.2 ± 1.8	< 0.0001	13.6 ± 3.1	7.8 ± 3.1	< 0.0001
Septal a’ (cm/s)	8.5 ± 2.1	8.2 ± 2.3	6.9 ± 1.8	0.2	9.7 ± 1.8	8.1 ± 1.7	< 0.05
Lateral a’ (cm/s)	8.8 ± 2.6	10.3 ± 2.5	6.1 ± 1.9	< 0.0001	8.7 ± 3.1	11.4 ± 2.1	< 0.01
Septal E/e’ ratio	8.8 ± 2.8	12.1 ± 5.0	17.4 ± 5.4	< 0.0001	8.6 ± 2.8	12.6 ± 6.4	< 0.01
Lateral E/e’ ratio	6.3 ± 2.1	7.8 ± 3.3	13.4 ± 5.0	< 0.0001	5.2 ± 3.1	8.1 ± 3.4	< 0.01

DD 1 and 2 indicates diastolic dysfunction grade 1 and 2, respectively, * p value is given for the comparison of Controls vs DD (ANOVA in the derivation group), values are given as mean ± SD, E and A, early (E) and late (A) mitral inflow velocity, IVRT indicates isovolumetric relaxation time, DT deceleration time, e’ and a’, tissue-Doppler derived early (e’) and late (a’) annular motion velocities.

### Electrocardiographic characteristics

**Figure 3:**
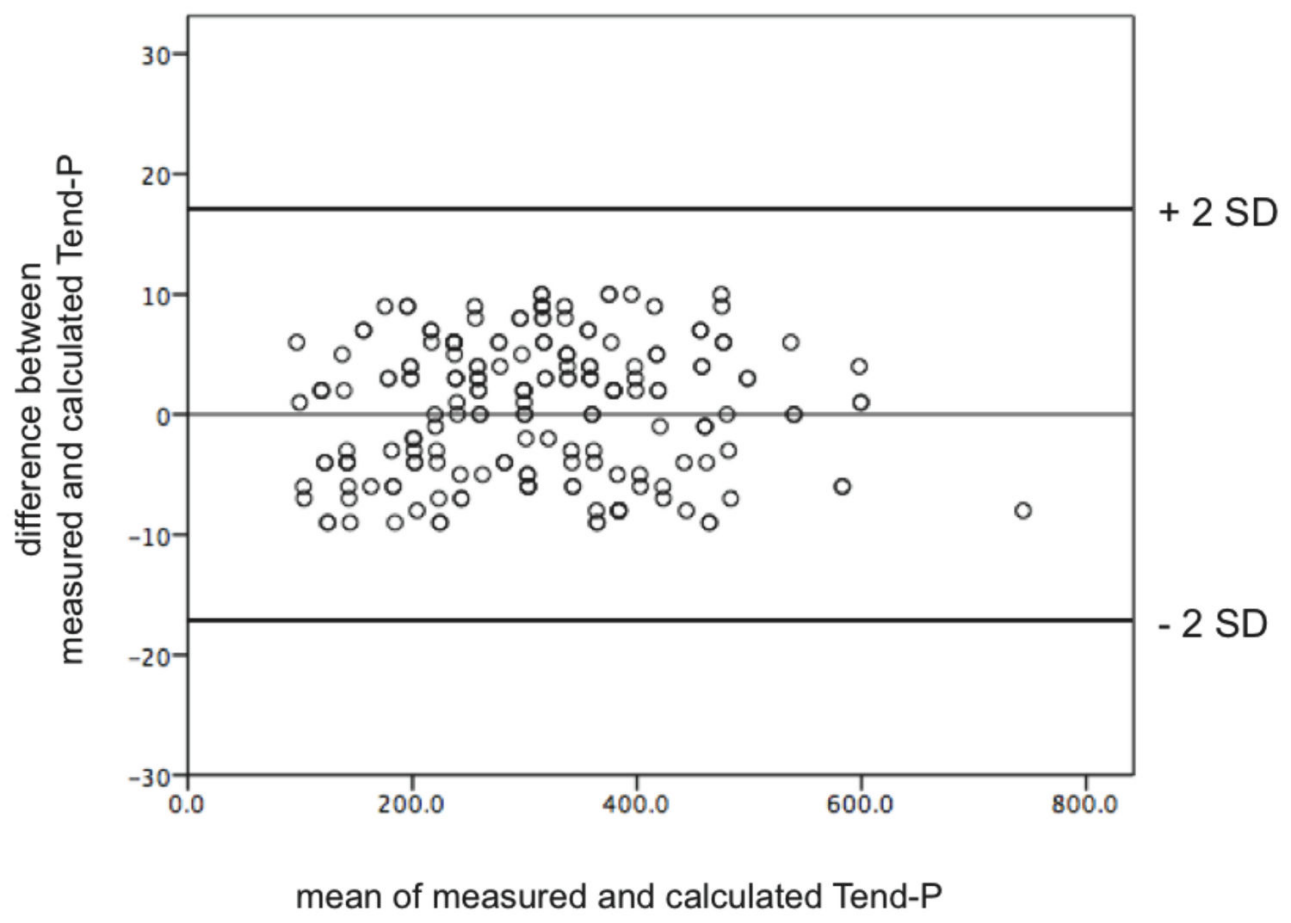
Bland – Altman Diagram for the correlation between manually measured and calculated Tend - P interval (ms). Manually measured diastolic intervals correlated perfectly with the calculated ones.

Electrocardiographic findings are summarized in Table 3. While patients in the derivation group showed a significantly higher heart rate than controls (78 ± 11 and 73 ± 12 vs. 67 ± 10 bpm, p<0.0001) there were no differences in heart rate between patients and controls in the validation group. Nevertheless, the QTc-intervals were longer in the patient group (422 ± 24 and 434 ± 32 vs. 409 ± 25 ms, p<0.0005). Furthermore, as expected, both Tend - P and Tend - Q intervals, reflecting the timing of the electrical as well as mechanical diastole, were shorter in the patient group (240 ± 78 and 276 ± 108 vs. 373 ± 110 ms, p<0.0001; 409 ± 85 and 447 ± 115 vs. 526 ± 119 ms, p<0.0001, respectively). Manually measured diastolic intervals correlated perfectly with the calculated ones (Figure 3, only shown for Tend - P). Moreover, PQ - intervals were significantly longer in the patient group (169 ± 28 and 171 ± 38 vs. 153 ± 22, p<0.005), which was also the case for the P wave duration (114 ± 19 and 110 ± 20 vs. 105 ± 18 ms, p<0.01), accounting primarily for the PQ - interval changes as the Pend - Q intervals were not significantly different. Of note, both the PQ - interval as well as P wave duration were longer in patients with DD despite their higher heart rate. Here again, most differences in the validation group were comparable to those of the derivation group (Table 3). 

**Table 3:** Electrocardiographic parameters.

		**Derivation Group**				**Validation Group**	
**Parameters**	**Controls**	**DD 1**	**DD 2**	p-value*	**Controls**	**DD 1**	p-value*
Heart rate (bpm)	67 ± 10	78 ± 11	73 ± 12	< 0.0001	71 ± 14	77 ± 12	0.1
Heart rate Echo (bpm)	69 ± 12	72 ± 9	67 ± 11	0.2†	69 ± 15	75 ± 15	0.7†
P wave duration (ms)	105 ± 18	114 ± 19	110 ± 20	< 0.01	101 ± 11	113 ± 21	< 0.0001
P wave amplitude (mV)	0.11 ± 0.04	0.11 ± 0.04	0.08 ± 0.03	0.1	0.11 ± 0.06	0.12 ± 0.06	0.1
PQ (ms)	153 ± 22	169 ± 28	171 ± 38	< 0.005	145 ± 22	167 ± 30	Q
Pend - Q (ms)	48 ± 21	54 ± 28	61 ± 38	0.09	44 ± 19	54 ± 23	0.07
P wave dispersion	50 ± 16	58 ± 16	62 ± 20	< 0.005	51 ± 17	57 ± 18	< 0.005
QRS (ms)	89 ± 12	90 ± 16	96 ± 14	0.2	88 ± 14	90 ± 13	0.7
QT (ms)	390 ± 33	371 ± 29	397 ± 38	< 0.005	388 ± 36	388 ± 30	1
QTc (ms)	409 ± 25	422 ± 24	434 ± 32	< 0.0005	416 ± 28	438 ± 32	< 0.05
Tpeak - Tend	69 ± 11	66 ± 12	72 ± 18	0.2	71 ± 14	69 ± 17	0.7
Tpeak - Tend dispersion	36 ± 14	35 ± 18	42 ± 15	0.4	38 ± 16	37 ± 17	0.6
QT dispersion	43 ± 17	44 ± 23	57 ± 33	0.09	45 ± 21	49 ± 23	0.3
QTc dispersion	46 ± 17	50 ± 26	64 ± 39	0.09	48 ± 16	49 ± 21	0.5
U wave (n/%)	27 (33)	22 (33)	7 (50)	0.8	15 (30)	16 (32)	0.9
Tend - P (ms)	373 ± 110	240 ± 78	276 ± 108	< 0.0001	349 ± 152	237 ± 131	< 0.0005
Tend - Q (ms)	526 ± 119	409 ± 85	447 ± 115	< 0.0001	494 ± 141	404 ± 129	< 0.0001

DD 1 and 2 indicates diastolic dysfunction grade 1 and 2, * p value is given for the comparison of Controls vs DD (ANOVA in the derivation group), values are given as mean ± SD or numbers and percentages, † p = ns for the comparison of heart rate values during echocardiographic vs. heart rate values during ECG in Controls and DD, respectively.

Subsequently, parameters differing significantly between the two derivation groups were correlated to the global assessment of diastolic function, after which five parameters remained significant: Age, Heart rate, PQ, Tend-P and Tend-Q. After adjustment for age, heart rate and PQ as possible confounders Tend-P and Tend-Q remained still significant (Table 4). Furthermore, we divided all individuals in two different age groups (Table 5) showing that differences in diastolic measures were not based on age-related relaxation abnormalities but “real” DD. However, none of these parameters showed a sufficiently high diagnostic performance (Table 6) why two novel indices combining the most relevant ones were generated. The underlying mathematical rationale was a more distinct differentiation of the assessed groups and thus a separation of overlaps by using the strongest single parameters (based on the above – mentioned analysis) and combing them: 

**Table 4:** Correlation and multivariate logistic regression analysis in the derivation group.

**Parameter**	p-value*	**Adjusted R^2^**	**p-value**
Age	< 0.0001		-
Heart rate	< 0.0001		-
P wave duration	0.8		-
PQ	< 0.0001		-
QT	0.3		-
QTc	0.9		-
P wave dispersion	0.8		-
Tend - P	< 0.0001	0.904	< 0.005†
Tend - Q	< 0.05	0.908	< 0.005†

* p value is given for the significance level of the multivariate correlation analysis

^†^ values are given for ANCOVA after adjustment for Age, Heart Rate and PQ

**Table 5:** Doppler-derived diastolic and electrocardiographic measurements in different age groups.

		**< 60 yrs**			**>60yrs**	
**Parameter**	**Normal**	**DD 1**	**DD 2**	**Normal**	**DD 1**	**DD 2**
Total Number	64	15	4	19	52	10
E/A	1.34±0.46	0.74±0.16	1.05±0.02*	1.05±0.02*	0.77±0.18	1.53±0.74*
DT	181±44	234±84	215±13†	208±35	230±55	187±28†
IVRT	86±29	87±24	98±25	103±30	94±25	86±20
Septal e’	8.8±2.4	5.2±1.0	4.9±1.4*	7.4±3.2	5.2±2.7	4.4±1.0†
Lateral e’	12.4±3.4	7.3±1.7	4.3±1.4*	9.4±2.8	8.2±3.2	6.9±1.5
Septal E/e’ ratio	8.4±2.7	9.9±2.9	16.5±6.2*	9.8±3.0	12.8±5.3	17.8±5.4†
LA ESVI	23.4±3.8	32.4±12.6	33.3±3.9*	23.3±3.8	33.4±10.8	40.6±12.1*
Tend - P / (PQ x Age)	0.060±0.026	0.0226±0.009	0.0269±0.005*	0.042±0.011	0.022±0.007	0.021±0.010*

DD 1 and DD 2 indicates diastolic dysfunction grade 1 and 2

* p<0.005 for the comparison of normal with DD (ANOVA)

^†^ p<0.05 for the comparison of normal with DD (ANOVA)

**Table 6:** Diagnostic performance values in the derivation and validation group for the recognition of diastolic dysfunction.

**Parameter (cut-off value)**	**AUC**	**Sensitivity**	**Specificity**	**PPV**	**NPV**	**Accuracy**
Age (≥58 years)	0.86	83%	75%	76%	82%	77%
PQ (≥150 ms)	0.65	78%	46%	58%	68%	62%
LA ESVI (≥34 ml/m^2^)	0.85	39%	100%	100%	56%	66%
Septal e’ (≤8 cm/s)	0.87	93%	56%	76%	83%	78%
Lateral e’ (≤10 cm/s)	0.84	85%	70%	80%	76%	79%
Septal E/e’ ratio (≥8)	0.79	83%	48%	71%	65%	69%
Septal E/e’ ratio (≥15)	0.79	34%	96%	93%	49%	59%
Tend - P (≤311 ms)	0.82	79%	72%	74%	78%	76%
Tend - Q (≤455 ms)	0.77	72%	73%	73%	73%	73%
Tend - P / (PQ x Age) (≥0.0333)	0.96	90%	92%	91%	90%	91%
Tend - Q / (PQ x Age) (≥0.0489)	0.95	89%	94%	94%	90%	91%
LAESVI & Tend - P / (PQ x Age)	0.98	96%	100%	100%	97%	98%
Sep E/e’ & Tend - P / (PQ x Age)	0.96	95%	100%	100%	95%	98%
**Validation Group**						
Tend - P / (PQ x Age) (≥0.0333)	0.91	82%	93%	93%	82%	88%
LAESVI & Tend - P / (PQ x Age)	0.95	90%	92%	95%	86%	91%
Sep E/e’ & Tend - P / (PQ x Age)	0.83	95%	57%	76%	89%	79%

AUC indicates the area under curve as assessed by ROC-Analysis, PPV positive predictive value, NPV negative predictive value

Tend–P/(PQxAge)Tend–Q/(PQxAge)

While both indices proved to have substantially higher diagnostic performance values as compared to traditional echocardiographic parameters (Table 6), the first index turned out to be slightly stronger, at a cut-off value of 0.0333 (DD < 0.0333 < normal). When combined with the indexed left atrial volume and the septal E/e’ ratio it showed a considerable added diagnostic value. 

### Validation of the novel index

**Figure 4:**
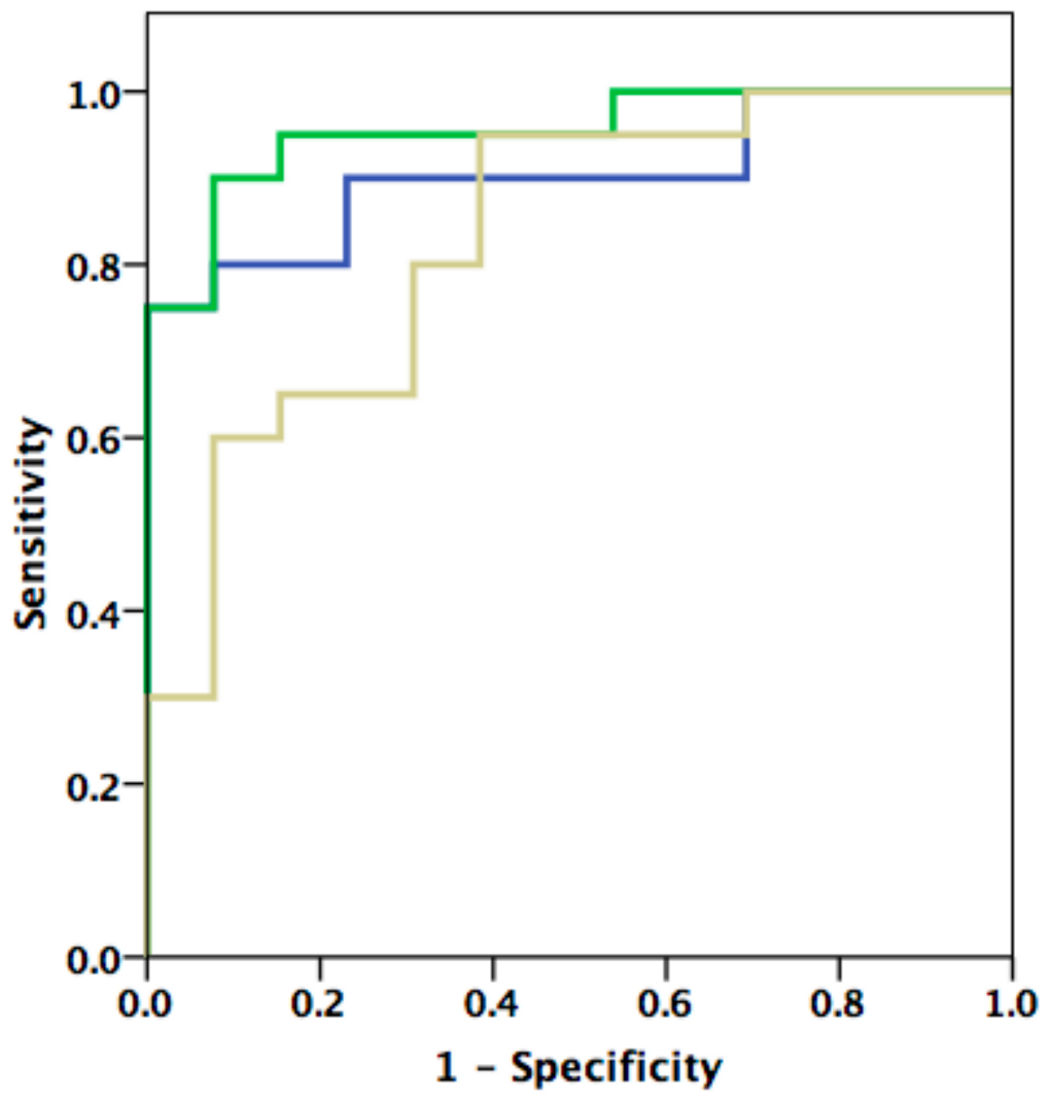
Receiver Operating Curve (ROC) analysis. ROC analysis showing diagnostic performance indices of echocardiographic parameters (LA-ESVI, septal E/e’) in combination with the electrocardiographic index Tend - P / (PQ x Age) in the validation group. See text and tables for values.

For further validation, we tested this index and its diagnostic performance at the evaluated cut-off value in the validation group. It showed a sensitivity of 82%, a specificity of 93%, a positive predictive value of 93%, a negative predictive value of 82% and an accuracy of 88% for the diagnosis of DD at the given cut-off value of 0.0333. Here again, the diagnostic performance for the recognition of DD was highest when combined with the indexed atrial volume (Table 6, Figure 4). 

## Discussion

To the best of our knowledge this is one of the first studies providing a systematic analysis of ECG indices and their value in the assessment of DD. Our main and novel findings are that

1. the PQ - interval and ECG indices reflecting the interval of mechanical diastole (Tend – P , Tend - Q) correlate very well with measures of DD.
2. a combined novel index consisting of Age, PQ - interval and Tend - P (Tend - P / [PQ x Age]) provides a substantial diagnostic performance, even after adjustment for possible confounders and when validated in an independent patient group.
3. the novel index shows a considerable added diagnostic value when combined with the indexed left atrial volume.

Different approaches are conceivable for the explanation of these observations. Generally, DD is associated with parameters mirroring functional and morphologic alterations including age and left atrial enlargement as well as left ventricular hypertrophy irrespective of the cause.

### P wave and PQ - interval in diastolic dysfunction and left atrial remodeling

 Left atrial dimensions are frequently viewed as a “barometer” of the chronicity of DD, which can reliably be deducted from ECG - based P wave and PQ - interval measures [3,14–16]. Furthermore, it has been shown that the PQ - interval is an exact determinant to define the timing of atrial contraction and thus atrial contribution to late diastolic left ventricular filling [17]. To that effect, as the PQ - interval lengthens, atrial contraction occurs earlier in diastole resulting in a shorter mid-diastolic slow ventricular filling and a shorter total diastole in patients with normal ventricular function, which has also been reflected by the shorter Tend - P interval in our patient group even after adjustment for heart rate [18]. Lengthening of the PQ - interval can also result in delayed and ineffective mitral valve closure and diastolic mitral regurgitation [19]. Our analysis showed a significant longer P wave duration as well as PQ - interval in patients with DD, which is in line with a recently published study [9]. Importantly, the differences observed in our study were not attributable to differences in heart rate. Besides, PQ - interval was twice as sensitive as the indexed left atrial volume (78% vs. 39%) for the recognition of DD, giving rise to the speculation whether changes in P wave / PQ - interval duration as an expression of left atrial remodeling processes might manifest earlier and more sensitive than respective morphological changes can be detected by imaging modalities. 

Prolongation of the PQ - interval can also occur in association with overt cardiovascular disease; however, this confounder is unlikely to explain our findings, since many of the individuals included in our study were generally healthy. Extensive discussions have focused on the question whether prolongation of the PQ - interval as such might also be indicative for general degenerative, i.e. age-related processes in the whole cardiovascular system contributing to a worse prognosis and/or emergence of cardiovascular comorbidities such as DD [20–22]. Accordingly, fibrosis and calcification of the cardiac skeleton after the age of 40 have been described already a few decades ago [23]. Thus, one of the early ECG manifestations of such processes might in fact be a PQ - interval prolongation. As a result, it does not seem counterintuitive that the same may be the case for the development of DD. Indeed, in both, age-related degenerative processes are amongst the major pathogenetic drivers or one of the common denominators for their pathogenesis and related cardiovascular risks and have been shown to be independently linked to adverse clinical events, such as the development of heart failure and atrial fibrillation [1,3,24–29]. Interestingly, the same has been repeatedly demonstrated for NT-proBNP and/or BNP measures. The latter further substantiates our findings and hypothesis, since BNP values were higher not only in the derivation but also in the validation group [30–32]. Nevertheless, we showed that our observations were not predicated on age-related gradual physiologic changes of LV diastolic function. These findings imply that prolongation of P wave duration as well as the PQ - interval as an indicator for left atrial remodeling and the development of DD may represent two distinct coincident phenomena without any causal relationship. However, further research may be necessary in particular to back up this matter. 

Besides, we found P wave dispersion to be more pronounced in patients with DD, another parameter, which has been described to have a certain association with remodeling processes of the left atrium and a higher risk for development of atrial fibrillation [33]. However, the value of P wave dispersion measurements as a predictor of atrial fibrillation is still a matter of debate. 

### Electrocardiographic depolarization and repolarization in diastolic dysfunction and left ventricular remodeling

Changes in left atrial dimensions most frequently come along with an elevated septal or posterior left ventricular wall thickness, eventually presenting as overt concentric left ventricular hypertrophy or remodeling. As these findings are very common and with rising prevalence in patients with hypertensive heart disease, they are one of the most frequently encountered abnormalities in patients with DD [1]. Left ventricular hypertrophy has been repeatedly reported in DD as well as in heart failure with a preserved systolic function by various groups [34,35], providing also histological evidence of considerable cardiomyocyte hypertrophy and a higher than normal left ventricular muscle mass [36]. Electrocardiographic parameters such as the prolongation of the QRS and QT/QTc interval are known to reflect an increased left ventricular muscle mass in patients with arterial hypertension [37,38]. Given the fact that only very few patients in our analysis presented with overt left ventricular hypertrophy, we did not find any differences in QRS duration. 

On the other hand, QTc intervals were longer in the patient group. This is in line with previous studies indicating a correlation between Doppler-derived parameters of DD and QTc duration [9,39]. The latter has also been extensively discussed in the literature for patients with inherited long - QT – Syndromes, where a relationship between a prolonged QT - interval and abnormal mechanical function was observed and supported by animal and cellular experiments [40–46]. Pathophysiologically, prolongation of the action potential duration may elicit manifest mechanical dysfunction through accumulation of intracellular calcium [47]. However, the correlation as well as diagnostic performance of the above-mentioned analyses were rather modest, possibly due to a certain degree of simplification by using only one electrocardiographic parameter. In fact, QTc prolongation may be caused by a prolongation of the Tpeak – Tend interval, as suggested by the same group in a more recent analysis [8]. Our analysis revealed a rather inferior (if not inexistent) correlation of the QTc and the Tpeak – Tend interval duration with echocardiographic measures of size, function and DD. The difference to previous studies can most likely be explained by the larger percentage of patients with a pseudonormal and/or restrictive filling pattern in the former, suggesting a more advanced cardiac disease stage and thus more pronounced repolarization alterations [8]. 

### Added diagnostic value of a combined approach

 Numerous noninvasive estimates of diastolic function and left ventricular filling pressures have been extensively investigated in the past decades including particularly Doppler-echocardiographic indices. However, many of them failed to yield a robust criterion for DD as a single parameter, why a comprehensive assessment of various indices and different algorithms have been proposed [1,2,48,49]. The latter analysis evaluated several echocardiographic measurements of diastolic function and the corresponding diagnostic performance at different cut-off values. While all measurements showed decent values for sensitivity and specificity, the indexed left atrial volume ≥ 34ml/min - being an independent predictor of atrial fibrillation, ischemic stroke, heart failure and death - turned out to be specific albeit less sensitive than other parameters [1,49,50]. In conclusion, a combination of lateral E/e’ ratio, indexed left atrial volume and the difference between duration of reverse pulmonary vein atrial systolic flow and duration of mitral valve atrial wave flow showed a considerable added diagnostic value with sensitivity and specificity values, which were similar to those in the strategy proposed by Paulus et al. [2], yet much higher than the one described by Nagueh et al. [1]. However, similar to other parameters, pulmonary vein flow measurements are known to be achievable only in a modest portion of all patients, thus depicting the limited applicability of certain Doppler-echocardiographic measurements [51]. While the latter confirms our experience, it has to be emphasized that the ECG parameters assessed in our analysis could be obtained in every individual. Furthermore, our combined strategy particularly including the novel index and the indexed left atrial volume yielded a substantial added diagnostic value for the recognition of DD, challenging the above-mentioned studies. 

### Limitations

Owing to the low number of patients with a DD grade 2 and 3, the patient group with a DD grade 1 is certainly overrepresented in the performed statistical analyses. However, this proportion mirrors the usual prevalences in the clinical setting. Nevertheless, we purposely showed demographic, echocardiographic and electrocardiographic parameters of the patient group with DD Grade 2 in order to depict congruent trends of the calculated mean values. Furthermore, matching groups in heart rate and age would have substantially lowered our population size and turned out not be entirely necessary as proven in the validation group. 

## Conclusion

The novel electrocardiographic index Tend–P/(PQxAge) shows a high diagnostic accuracy for the diagnosis of DD and yields a substantial added value when combined with the indexed left atrial volume. Our findings add substantially to the growing evidence of a probable interplay of structural remodeling processes and electromechanical coupling in the pathogenesis of DD. 
